# Intentional Switching Between Bimanual Coordination Patterns in Older Adults: Is It Mediated by Inhibition Processes?

**DOI:** 10.3389/fnagi.2020.00029

**Published:** 2020-02-18

**Authors:** Jean-Jacques Temprado, Marta Maria Torre, Antoine Langeard, Marine Julien-Vintrou, Louise Devillers-Réolon, Rita Sleimen-Malkoun, Eric Berton

**Affiliations:** ^1^Aix-Marseille Université and CNRS, UMR 7287 Institut des Sciences du Mouvement Etienne-Jules Marey, Marseille, France; ^2^Università degli Studi di Roma “Foro Italico,” Rome, Italy; ^3^Department of Medicine, University of Montreal, Montreal, QC, Canada; ^4^Research Centre, Montreal Heart Institute, Montreal, QC, Canada; ^5^Centre de Recherche, Institut Universitaire de Gériatrie de Montréal, Montreal, QC, Canada

**Keywords:** aging, bimanual coordination, switching, Stroop task, inhibition, mediation analysis

## Abstract

The study investigated the consequences of age-related decline in inhibition processes on intentional switching between bimanual coordination patterns. Fifteen young (24±2.8 years) and 20 older adults (69±5.3 years) performed Stroop tasks and bimanual coordination tasks. Stroop tasks included neutral, congruent, and incongruent conditions. Response time and error rate were measured. Bimanual coordination tasks consisted of performing in-phase (IP) and anti-phase (AP) patterns. Participants were requested to switch as quickly as possible from one pattern to the other, resulting in two different switching directions (AP to IP; IP to AP). Mean and standard deviation (SD) of the continuous relative phase (CRP) were calculated pre- and post-switching for each participant. Total switching time (TST) was measured. The switching phase was also decomposed into reaction time (RT) and reversal time (REvT). Pearson correlation analyses were performed to test for correlations between: (i) SD of CRP and response time in Stroop tasks, and (ii) switching times (TST, RT, RevT) and response time in Stroop task, respectively. In addition, parallel mediation analyses were conducted. Results showed that: (i) the AP pattern was less stable than the IP pattern in both young and older adults, (ii) coordination patterns were less stable in older adults, (iii) response times in Stroop task were longer in the incongruent condition, and (iv) RespTs were longer in older than in young participants, whatever the condition. In the bimanual coordination task, RT, RevT, and TST increased with age. The stability of the IP pattern was correlated with the response times observed in neutral and congruent conditions, while the stability of the AP pattern was correlated with response time observed in the incongruent condition. Correlation and mediation analyses showed that, in the AP to IP switching direction, RT and RevT were both significantly correlated with response times observed in the incongruent condition of Stroop task. These findings suggest that inhibition processes are involved in switching between bimanual coordination patterns, at least to trigger the early phase of switching. They also support the hypothesis that inhibition processes are more involved in maintaining the AP pattern and switching to the IP pattern. Finally, age-related changes in switching times seem to be prominently mediated by alterations of inhibition processes.

## Introduction

The quality of everyday life of older adults depends to a large part on their ability to carry out coordinated movements and, in particular, bimanual coordination (e.g., dressing yourself, tying shoelaces, lifting and carrying objects, eating, or typing an email). Therefore, understanding the impact of aging on bimanual tasks is important for preserving independence and well-being in older adults.

Cognitive-motor interactions are of particular interest in this respect. Indeed, aging strongly alters cognitive processes [and in particular executive functions (EFs)], which are in turn more and more involved in the control of complex movements during aging (e.g., Heuninckx et al., 2005, 2008; Coxon et al., 2010). Accordingly, during the last 10 years, much attention has been drawn to the understanding of neural and cognitive underpinnings of bimanual coordination in young (e.g., Temprado et al., 1999; Swinnen, 2002) and older adults (e.g., Lee et al., 1996; Heuninckx et al., 2005; for an overview, see Maes et al., 2017). However, whether and how age-related alterations of EFs affect behavioral adaptability in bimanual coordination tasks is still unclear. Indeed, cognitive modulation of the stability of existing coordination patterns has been extensively investigated in young adults (Scholz and Kelso, 1990; Lee et al., 1996; Byblow et al., 1999; Serrien et al., 1999; Temprado et al., 1999; Poel et al., 2006; De Luca et al., 2010; Tallet et al., 2010; Maslovat et al., 2017; Wang et al., 2018) but scarcely in older adults (see Greene and Williams, 1996; Coxon et al., 2010 for noticeable exceptions). The present study addresses this issue in the theoretical context of Coordination Dynamics (Kelso, 1995, 2002).

At the core of this approach is the assumption that, in bimanual cyclic tasks, the two hands can be seen as self-sustained oscillators linked through a non-linear coupling function. As a consequence, stable preferred patterns and spontaneous transition between them emerge from the coalition of multiple constraints that arise within and between the different subsystems and components of the organism (Haken et al., 1985; Kelso, 1995, 2002). Accordingly, bimanual coordination is characterized by two preferred patterns [i.e., in-phase (IP) and anti-phase (AP)] (Kelso, 1984), which are captured by the values of relative phase (RP) between the two hands, while their stability is indexed by the magnitude of fluctuations of RP (SD of RP) (Haken et al., 1985). By convention, the IP pattern refers to bimanual coordination that results from the simultaneous activation of homologous muscle groups, thereby giving rise to mirror symmetrical movements with respect to the body midline. Conversely, the AP pattern refers to bimanual coordination that results from simultaneous activation of non-homologous muscle groups, so that one limb moves toward the body midline, while the other limb moves away from it and vice versa. Accordingly, it has been shown that: (i) the IP pattern is more stable than the AP pattern, and (ii) when intentional modulation is minimal (the “do not intervene” instruction), an unavoidable switch from the latter to the former occurs when oscillation frequency increases beyond a given critical threshold (e.g., Kelso, 1984; Temprado et al., 2010). Thus, behavioral stability plays a central role in the behavioral dynamics of bimanual coordination (Haken et al., 1985; see also Kelso, 1995, for a detailed description).

However, the constraints imposed by spontaneous coordination dynamics on the actual behavior do not preclude flexibility. Indeed, several studies have shown that participants may intentionally: (i) delay or inhibit the spontaneous transition from one pattern to another (Lee et al., 1996); (ii) momentarily stabilize an existing preferred coordination pattern (Swinnen, 1996; Amazeen et al., 1997; Riley et al., 1997; Temprado et al., 1999); (iii) permanently stabilize a novel coordination pattern with learning (Zanone and Kelso, 1992, 1997), and (iv) intentionally switch between preferred coordination patterns (Scholz and Kelso, 1990; Carson et al., 1996; Byblow et al., 1999). In all these situations (i.e., stabilizing, learning, intentional switching), cognition represents an intervening variable in the (de)stabilization of behavioral patterns. Indeed, by definition, intrinsic dynamics accounts only for spontaneously stable states (i.e., patterns that emerge without any extraneous influence directly affecting RP), while by contrast, intention stipulates a stable state of the coordination dynamics that attracts RP to a given value. For instance, intentional switching may be viewed as a supplemental force cooperating with the “spontaneous” coordination dynamics to selectively de- or re-stabilize the IP or AP pattern (Schöner and Kelso, 1988). In this context, it is currently considered that intentional switching between coordination patterns involves inhibition processes. However, the consequences of age-related cognitive decline (in particular EFs and inhibition process) on intentional switching have been scarcely investigated. The present study is a step in this direction.

It is well established that aging alters the stability of bimanual coordination patterns (Bangert et al., 2010; Krehbiel et al., 2017; Maes et al., 2017) and their dynamics (Temprado et al., 2010). Specifically, older adults are more variable than young adults and, as a consequence, they show decreased critical transition frequency (Temprado et al., 2010). Also, aging makes bimanual coordination more cognition-dependent (Heuninckx et al., 2005, 2008; Maes et al., 2017). For instance, performing bimanual coordination patterns incurs more attentional load (Lee et al., 2002; Sparrow et al., 2005) and requires more executive control in older adults than in young participants (Heuninckx et al., 2005, 2008; Bangert et al., 2010; Goble et al., 2010). Heuninckx et al. (2005, 2008) observed that older adults exhibited additional activation of specific brain structures (anterior rostral cingulate and dorsolateral prefrontal cortex) that are involved in suppressing spontaneous response tendencies and inhibitory cognitive control. Consequently, it can be hypothesized that age-related decline in global cognition (i.e., EFs, information processing efficiency) and, more particularly, inhibition processes, should have consequences on intentional switching between bimanual coordination patterns. The present study explored this hypothesis by investigating whether inhibition processes mediate intentional switching capacities between bimanual coordination patterns in young and older adults.

The paradigm of intentional switching between bimanual coordination patterns has been introduced by Scholz and Kelso (1990) to test the hypothesis that switching time from one pattern to another depends on the differential stability of the IP and AP patterns. In this study, participants were instructed to move both index fingers rhythmically at different movement frequencies while performing either an IP or AP coordination pattern. On cue from an auditory signal, they switched from the ongoing pattern to the other pattern. Results showed that switching from IP to AP was significantly slower than switching in the opposite direction. Thus, intentional switching was influenced by the initial stability of the ongoing pattern. However, although RP fluctuations increased when moving in the AP pattern at the highest movement frequencies, movement frequency had little effect on switching time. Scholz and Kelso (1990) concluded that cognition (intention) allowed modifying the intrinsic dynamics of coordination patterns, while these dynamics influenced, in turn, the resulting behavior. These results were confirmed by Carson et al. (1996), who also reported that switching time decreased in a linear fashion with increasing movement frequency (for consistent results, see De Luca et al., 2010; Carter et al., 2017; Wang et al., 2018).

Brain imaging studies have shown that intentional switching is mediated by specific neural structures. For instance, using functional magnetic resonance imaging to explore the role of basal ganglia (BG), De Luca et al. (2010) showed greater activity in bilateral putamen when subjects were required to switch from a more stable (IP) to a less stable (AP) pattern than vice versa. Thus, the role of putamen could be to select desired actions and inhibit competing ones through parametric modulation of the intrinsic dynamics. Kelso (2012) formalized the interaction between intention and intrinsic dynamics on the basis of BG activity. According to this modeling hypothesis, less BG activity would be required to switch for the AP pattern to the IP one since the transition is facilitated by the intrinsic dynamics. Conversely, more activity would be required to select the IP pattern since the intrinsic dynamics should be forced to intentionally trigger the transition (for details, see Kelso, 2012, p. 910). Thus, according to De Luca et al. (2010) and Kelso (2012), the less stable the ongoing pattern, the lower the load on inhibition processes (and the BG activity) during switching. In this respect, one can suppose that during spontaneous transition the related neural network is very slightly involved, since in the spontaneous transition is a spontaneous response that let it to happen, in comparison with intentional one.

However, this conclusion can be challenged on the basis of existing literature on bimanual coordination switching in older adults. Indeed, if intentional switching was only related to pattern stability, switching time should be shorter in older adults than in young participants, at least for the AP pattern, since coordination patterns are less stable in older adults than in young participants (e.g., Temprado et al., 2010). However, results reported in the literature showed that switching time was longer in older adults for both switching directions (i.e., AP to IP; IP to AP) (Greene and Williams, 1996; Coxon et al., 2010). This result suggests that, although older adults exhibit less stable coordination patterns, their ability to suppress and flexibly adapt coordination behavior (i.e., to switch between patterns) declines. Thus, spontaneous phase transitions seem to obey different principles than intentional transitions. Indeed, spontaneous transitions are easier to induce in older adults since the AP pattern is less stable (Wishart et al., 2000; Heuninckx et al., 2004; Temprado et al., 2010), but intentional transition between AP to IP takes longer in older adults despite the lower stability of coordination patterns. A possible reason is that when switching between coordination patterns, compared with young adults, the elderly show less engagement of interconnected BG and SMA structures, which are critical for inhibitory control of action (Coxon et al., 2010; De Luca et al., 2010). In compensation, they rely more on frontal cortical structures (i.e., EFs) and switching time increases (Coxon et al., 2010).

### Aim and Hypotheses of the Study

In the present study, we systematically investigated the relation between intentional switching and inhibition processes in young and older adults: (i) by analyzing the effects of the direction of transition, movement frequency, and aging on switching times; (ii) by analyzing the mediation of inhibition processes on the intentional transition from one pattern to the other. Our general hypothesis was intentional switching times would be influenced by the direction of the transition (i.e., AP to IP shorter than IP to AP), movement frequency (low frequency longer than high frequency), and age (young shorter than older). To test this hypothesis, we measured the total switching time (TST) that is the time required to switch from the ongoing pattern to stabilize the targeted pattern (see section “Materials and Methods” for a detailed description). In addition, we also decomposed switching time into subparts, which presumably are differentially sensitive to the different factors, namely: (i) reaction to the signal (RT), which measures the time required to start dismantling the ongoing pattern; (ii) hand reversal time (RevT), which refers to the time of occurrence of hand reversal movement toward the new pattern.

In addition, particular attention was given to the increasing role of inhibition processes in bimanual switching with age. Specifically, we expected that: (i) switching would be even more difficult in older adults due to the alteration of inhibition processes and (ii) this should be more marked for the AP pattern since presumably, inhibition processes are more involved in AP pattern than in IP one.

However, based on the theoretical framework of coordination dynamics, we also examined the alternative hypothesis that the intrinsic dynamics could play a prominent role in the switching process (Kelso, 2012). In this case, switching reaction time (RT) would be faster in the older adults, at least for AP to IP, due to the lower stability of the AP pattern.

## Materials and Methods

### Participants

Fifteen young adults and 20 older adults voluntarily participated in the experiment (mean age 24±2.8 and 69±5.3 years, respectively). Young participants were students of Aix-Marseille University, while the older participants were recruited after a call on the local newspaper. The protocol was approved by the local ethics committee of Aix-Marseille University and is in accordance with the ethical standards laid down in the Declaration of Helsinki.

### Inclusion Criteria

The inclusion criteria were: (i) not suffering from hand, arm, or shoulder impairment (e.g., arthritis), (ii) not having a surgery in the past 6 months, (iii) not taking medications known to affect cognition, and (iv) having a normal vision and hearing. In addition, participants completed the MoCA test (Nasreddine et al., 2005), which allowed testing different functions (attention, concentration, EFs, memory, language, visual-constructive abilities, abstraction, calculation, orientation). To be included in the study, participants had to have a score greater than 26.

### Task and Apparatus

#### Stroop Task

Participants were seated on a chair in front of a computer screen and a modified keyboard with only four adjacent keys. Words were presented on the screen and were written in a specific color (green, blue, red, or magenta). For instance, the word green was printed either in green or in red (translated in French since participants were French native). Participants were requested to indicate the color in which the word was written by pressing as quickly as possible on the corresponding key of the keyboard (G when written in green, R when in red, etc.), while inhibiting the word’s semantic. Thus, depending on the consistency between the word and the color, the trial was considered congruent (C, e.g., the word “green” written in green) or incongruent (I, the word “green” written in red). In other trials, “neutral” words (N) were presented (e.g., arm, leg…), which were written in one of the different colors. Sixty words (20 C, 20 N, and 20 I) were presented (5 for familiarization + 55 for testing). We measured response times that is the time elapsing between the appearance of the word on the screen and manual pressing the key on the keyboard. The word remained on the screen until the response was given.

##### Bimanual coordination

**FIGURE 1:**
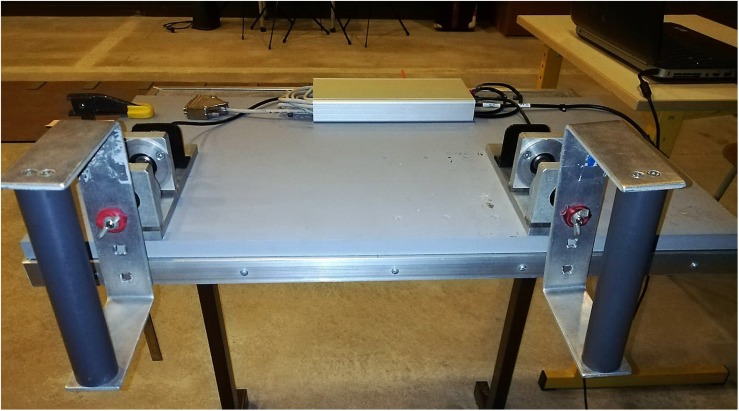
Experimental setup.

The experimental setup was the same as the one used by Temprado et al. (2010) (see Figure 1). Participants were seated on a chair and leaned with their back against the chair support to prevent shoulder movements. Their upper limbs were placed parallel to each other. Flexion angles of elbows were about 90°. Participants gripped free rotating handles with their fingers flexed, thereby allowing them to easily perform pronation-supination movements of the forearms in order to move the handles. The axis of rotation of each handle was positioned between digits III and IV of each hand. The width between the handles could be adjusted depending on the participants to adopt the most comfortable position. Each handle could rotate within a 90° range. This large amplitude range allowed participants to have virtually unrestricted movements of their forearms. Handle movements were recorded at a sampling frequency of 100 Hz using two potentiometers placed, respectively, on the axis of rotation of each handle.

Participants were requested to perform IP and AP bimanual coordination pattern by making forearm pronation-supination movements in the frontal plane in synchrony with a metronome that prescribed the required frequency in the course of trials (see Temprado et al., 2010 for a similar procedure). By convention, patterns resulting from simultaneous activation of homologous muscles—giving rise to mirror-symmetric movements with respect to body midline—were denoted IP. Coordination patterns resulting from simultaneous activation of non-homologous muscles—giving rise to asymmetric movements—were denoted AP.

### Procedure

After having completed the MoCA test and the Stroop task, participants performed the bimanual coordination task. Before the experiment, participants were informed about the procedure and the tasks they will have to perform. Then, a period of familiarization with coordination patterns was proposed, during which they performed each pattern for 2 min at a freely chosen frequency. They learned to make continuous oscillatory movements with a large amplitude (i.e., at least ±45° around the central position). Participants performed two blocks of trial, which allowed: (i) identifying the transition frequency and (ii) investigating intentional switching.

#### First Block of Trials—Identification of the Transition Frequency

Participants were requested to perform the AP pattern while following the frequency given by the metronome. They were instructed: (i) to perform one full cycle of movement between two beats of the metronome, and (ii) to keep the amplitude of movements of each hand in a range of ±45° around the central position. In the course of trials, the experimenter gave feedback to the participants on whether the movement needed to be increased in amplitude or to be maintained with an equal amplitude adjusted in both directions around the central position. In young participants, the metronome frequency was increased by 0.5 Hz every 10 s, starting from the baseline of 1 Hz until a maximum of 3 Hz. In older participants, the procedure was adapted: frequency was set from 1 to 2 Hz with steps of 0.25 Hz every 10 s. Participants were instructed to move hands without intervening, and let the pattern switching whenever they were feeling to lose the AP relationship. Transition frequency was defined as the frequency at which the participants switched from AP to IP. Five trials were performed in order to reliably detect the mean transition frequency (MTF).

In the subsequent block of trials, MTF of each participant was then decreased by 0.25 Hz, and the resulting frequency value was used as the highest frequency for investigating intentional switching. In older adults, two participants exhibited a phase transition at 1.25 Hz so that the lower frequency was set at 0.75 Hz and the high frequency at 1 Hz.

#### Second Block of Trials—Intentional Switching

Four blocks of 10 trials (30 s each) were randomly presented to the participants of both age groups. They resulted from crossing patterns (IP, AP) and frequencies (low, high). The low frequency was fixed at 1 Hz, and the high frequency corresponded to MTF -0.25 Hz. In each block of trials, when the tone of the metronome changed, participants were requested to switch as quickly as possible from the current pattern to the target one. Thus, the switching task was presented to participants as an RT task, in which they must destabilize the current pattern and stabilize the target one as fast as possible. Change in signal tonality was randomly presented between the 15th and the 18th second of the trial. For the blocks of trials starting IP, both forearms were placed in pronation for five trials and in supination for the other five trials. For the blocks of trials starting AP, in five trials, the left forearm was placed in pronation and the right in supination, while in the five other trials, the right forearm was placed in pronation and the left in supination. This procedure allowed randomizing the position of the forearms at the onset of the switching signal.

### Data Reduction and Analysis

#### Stroop Task

The raw data were processed with a customized MATLAB routine (MathWorks Inc., Natick, MA, United States). The number of errors was calculated for each trial condition (congruent, incongruent, neutral). Response time of each correct trial condition was then calculated and averaged for each participant. Response times values under or below two times standard deviation (±2 SD) of each participant mean were considered as outliers and deleted. On the basis of this criterion, over the 55 trials performed, two trials in the group of young participants and three in the older adults were discarded. The percentage of errors in congruent and neutral conditions was about 2% and about 10% in the incongruent condition.

#### Bimanual Coordination Data Analysis

The raw data were processed with a customized MATLAB routine (MathWorks Inc., Natick, MA, United States). They were filtered with a Butterworth filter (cut-off frequency 10 Hz, order 2). Then, amplitude centering procedure was used to remove frequency artifacts of the non-sinusoidal signals, when existing. According to Lamb and Stöckl (2014, Eq. 4), the normalization was based on the function:

$$g\,\left(y\,\left(t\,\text{i}\right)\right)=\,2\,\,\frac{(\text{y}\left(t\text{i}\right)-\min\left(y\left(t\right)\right)}{\max\left(y\,t\right)-\min\left(y\,\left(t\right)\right)}-1$$

This function transforms the original values y(t) in such a way that the minimum value of g(y(t)) equals -1 and the maximum value of g(y(t)) equals 1.

To calculate the continuous RP (CRP), a new signal generated representing the difference in phase angles of the two original signals (Lamb and Stöckl, 2014), we first calculated the phase of each hand on the basis of normalized phase portraits. Subsequently, CRP was calculated after the application of Hilbert transform sign the following formula:

$$C\,R\,P\,\,\left(t_{i\,}\right)=\,C\,R\,P_{l\,e\,f\,t}\,\left(t_{i\,}\right)$$

$$-\,C\,R\,P_{r\,i\,g\,h\,t}\,\left(t_{i\,}\right)\,\,a\,r\,c\,t\,a\,n\,\left(\frac{H_{1}\,\left(t_{1}\right)\,x_{2}\,\left(t_{1}\right)-\,H_{2}\,\left(t_{1}\right)\,x_{1}\,\left(t_{1}\right)}{x_{1}\,\left(t_{1}\right)\,x_{2}\,\left(t_{1}\right)-\,H_{21}\,\left(t_{1}\right)\,H_{2}\,\left(t_{1}\right)}\right)$$

Then, we calculated the mean and SD of the CRP for each participant. After calculation, the times series of CRP were divided into pre-switching and post switching phases (Lamb and Stöckl, 2014). For the pre-switching phase, we calculated the mean and SD of CRP.

**FIGURE 2:**
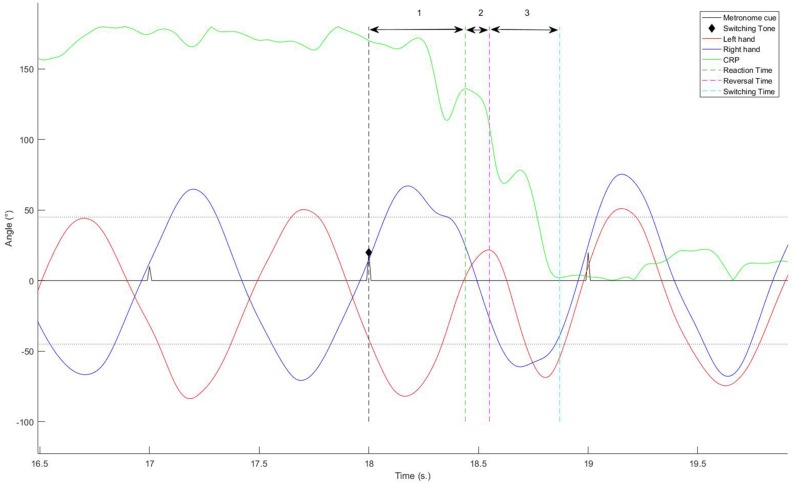
Decomposition of the switching phase into different sub-parts.

The TST was defined as the time lapsing between the switching signal and the first mean value of CRP post-transition that preceded at least 3 s of stabilization within a range of ±45° around the CRP value corresponding to the requested pattern (i.e., either IP or AP). In addition, the switching phase was decomposed into different sub-parts, and the duration of each of them was calculated (see Figure 2):

• *RT:* the interval between the signal of changing given by the metronome the first value of RP outside of ±45° of the value corresponding to the currently performed pattern.
• *RevT*: the interval between the signal and the first reversal movement of one hand, allowing to gain half a cycle (or less) to switch to the new pattern. For each trial, we also identified the hand that triggered the switching.

The TST was calculated for the trials where the CRP remained for at least 3 s in a range of ±45° of the targeted CRP during post-switching (e.g., ±180° for the AP pattern and ±0° for the IP pattern). When a trial did not meet this criterion, it was considered as an outlier and excluded from the analysis. On average, for each participant, a maximum of one trial per block was excluded for both age groups.

### Statistical Analysis

For the Stroop task, we carried out a 2 age × 3 condition ANOVA on the response times. For the bimanual coordination, we carried out 2 age × 2 frequency × 2 pattern ANOVA on the SD of CRP and the TSTs, RTs, and RevTs. The chosen level of statistical significance was *p* < 0.05. Then Pearson correlation analyses were performed to test for correlations between: (i) SD of RP and response times in Stroop task, and (ii) the different switching times in the bimanual coordination task, and response times in Stroop task, respectively.

Finally, parallel mediation analyses were conducted with comparable methods of previous protocols (e.g., Langeard et al., 2019). Parallel mediation allowed studying the relationship between one independent variable (X), one dependent variable (Y), and more than one simultaneous mediator variable (M_n_). In the present study, six parallel mediation analyses were conducted for six dependent variables (Y) corresponding to the three switching outcomes in both directions. The independent variable was the age group, and three mediators corresponding to the three Stroop conditions were used simultaneously. This allows isolating a specific Stroop condition effect while controlling for the others. The effects of X on M_n_ are called the “a_n_” paths, and the effects of M_n_ on Y while controlling for X are called the “b_n_” paths. The amount of mediation is called the “indirect effect.” The indirect effects (a_n_b_n_) refers to the role of each particular mediator (M_n_) in the relationship between X and Y. We tested the significance of the indirect effects using bias-corrected bootstrap confidence intervals (CIs) (based on 5000 bootstrap samples in the present study). CI that did not contain zero represent significant effects, and therefore significant mediation of X on Y through M. The centrality and normality of the residuals were verified. Analyses were performed using IBM SPSS Statistics (IBM Corp., Armonk, NY, United States), with the PROCESS macro for mediation analyses (Hayes, 2017).

## Results

### Stroop Task

The ANOVA performed on response times revealed a significant main effect of age [*F*(1,62) = 55.133, *p* < 0.01] and condition [*F*(1,62) = 10.44, *p* < 0.01]. Older adults were slower than young ones. The *post hoc* decomposition of the condition effect using the Newman–Keuls revealed a significant difference between congruent and neutral conditions with the incongruent one, but not between congruent and neutral ones (*p* > 0.05).

### Bimanual Coordination—Identification of MTF

Mean transition frequency and the frequencies finally used for each participant in the switching experiment (MTF -0.25 Hz) are represented in Table 1.

**TABLE 1:** MTF—0.25 Hz in both the age group ranked from the lower to the higher.

Participants young	MTF—0.25 (Hz)	Participants old	MTF—0.25 (Hz)
1	1.25	1	1
2	1.25	2	1
3	1.5	3	1.25
4	1.5	4	1.25
5	1.5	5	1.25
6	1.5	6	1.25
7	1.75	7	1.25
8	1.75	8	1.25
9	1.75	9	1.25
10	1.75	10	1.25
11	2	11	1.25
12	2	12	1.25
13	2	13	1.25
14	2.25	14	1.25
15	2.25	15	1.25
		16	1.25
		17	1.5
		18	1.5
		19	1.5
		20	1.5

*MTF, mean transition frequency.*

### Bimanual Coordination—Intentional Switching

#### SD of RP

The values of SD of RP observed during pre-switching were submitted to an age × pattern × frequency ANOVA. This analysis revealed a significant effect of age [*F*(1,131) = 18.52, *p* < 0.01], direction [*F*(1,131) = 34.27, *p* < 0.01)], and frequency [*F*(1,131) = 28.81, *p* < 0.01]. The age × frequency [*F*(1,131) = 4.6, *p* < 0.05] and pattern × frequency [*F*(1,131) = 6.27, *p* < 0.05] interactions effects were also found significant. The *post hoc* decomposition of the age × frequency interaction using the Newman–Keuls test showed that frequency had no effect on SD of RP in older adults. In addition, the decomposition of the pattern × frequency interaction showed that frequency had no effect on the IP pattern. Accordingly, we decided to merge the two frequencies for the subsequent analyses.

Accordingly, the age × pattern ANOVA carried out on the SD of RP revealed a significant main effect of age [*F*(1,70) = 8.36, *p* < 0.01] and pattern [*F*(1,70) = 26.69, *p* < 0.01]. Specifically, older participants were more variable than young participants (9.75° and 8.75°, respectively). The SD of AP pattern was larger than the SD of the IP pattern (10.45° and 7.3°).

#### Switching Times

The age × pattern ANOVA showed that older adults had a significant longer TST [*F*(1,66) = 19.03, *p* < 0.001], longer RT [*F*(1,66) = 10.7, *p* < 0.001], and longer RevT [*F*(1,66) = 14.3, *p* < 0.001] than young adults. Moreover, TST from IP to AP was significantly longer than switching from AP to IP [*F*(1,66) = 5.73, *p* < 0.05] (Table 2).

**TABLE 2:** Mean and SD of TST, RT, and RevT (in milliseconds) for the different switching directions in young and older adults.

		Young		Old	
		Mean	*SD*	Mean	*SD*
TST	IP to AP	1226	372	1608	467
	AP to IP	885	93	1417	522
RT	IP to AP	482	142	608	139
	AP to IP	475	96	538	90
RevT	IP to AP	593	139	725	145
	AP to IP	573	101	662	86

*TST, total switching time; RT, reaction time; RevT, reversal time; IP, in-phase; AP, anti-phase.*

### Correlations

Significant correlations between SD of RP and switching with Stroop variables were detected for several conditions.

**FIGURE 3:**
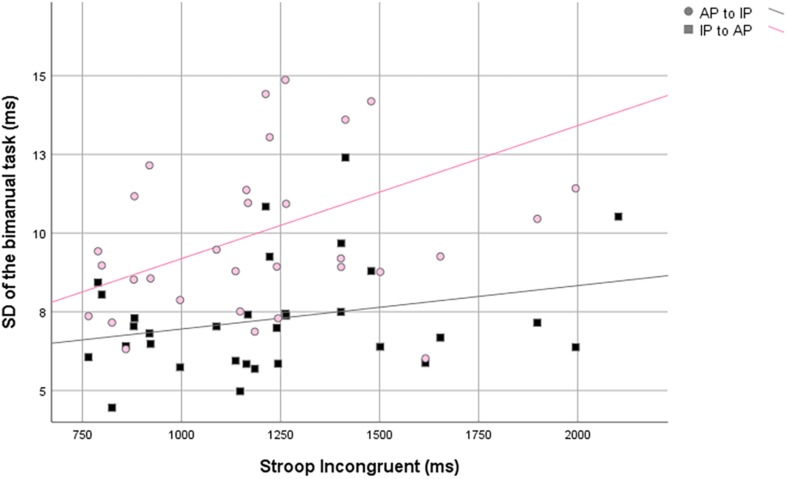
Scatter plots of the standard deviation of the relative phase against the Stroop incongruent condition.

First of all, in the IP to AP direction, SD of RP (i.e., of the IP pattern) was positively correlated with the RT of the congruent and, moreover, with the neutral condition of Stroop task. In the AP to IP direction, SD of RP (i.e., of the IP pattern) was positively correlated with all three conditions of Stroop task (Table 3 and Figure 3).

**TABLE 3:** Correlation analysis between SD of RP and the responds time in the three conditions Stroop task.

SD of RP	RT congruent	RT neutral	RT incongruent
IP to AP	0.414*	0.466**	0.276
AP to IP	0.579**	0.561**	0.463**

* and ** indicate the significant correlations (0.05 and 0.01, respectively). RT, reaction time; IP, in-phase; AP, anti-phase.

Second, RT and RevT measured during the bimanual switching task, in both AP to IP and IP to AP directions, were correlated with incongruent RTs measured in the Stroop task. Moreover, TST measured in the AP to IP direction was correlated to both congruent and neutral RTs of the Stroop task. Finally, RevT measured in the AP to IP direction was correlated to congruent RT of the Stroop task (Table 4).

**TABLE 4:** Correlation analysis between switching times and responds times in the three conditions of Stroop task.

		RT congruent	RT neutral	RT incongruent
TST	IP to AP	0.340	0.338	0.276
	AP to IP	0.426*	0.469**	0.293
RT	IP to AP	0.172	0.137	0.359*
	AP to IP	0.333	0.207	0.453**
RevT	IP to AP	0.188	0.171	0.365*
	AP to IP	0.404*	0.338	0.491**

* and ** indicate the significant correlations (0.05 and 0.01, respectively). TST, total switching time; RT, reaction time; RevT, reversal time; IP, in-phase; AP, anti-phase.

### Mediations Analysis

The three “a_n_” paths were significant. They revealed an effect of age on the three Stroop conditions with large size effects (Table 5). Among the “b_n_” paths predicting the switching scores from the three Stroop conditions, only the paths going through the incongruent Stroop condition were significant. Specifically, RT from IP to AP, RT from AP to IP, and RevT from IP to AP were predicted by response times in the Stroop incongruent condition with an important effect size (Table 6). Finally, bootstrap analyses of the indirect “a_n_b_n_” paths detected a significant mediation with a large effect size of RT in the AP to IP condition through the response time of the incongruent Stroop condition (Table 7). Altogether, these results show that the relationship between age group and RT from AP to IP is mediated by the RT of the Stroop incongruent condition while controlling for the other Stroop conditions.

**TABLE 5:** Relation a_n_ in the mediation analysis.

	Coeff	SE	*T*	*p*	Standardized
RT congruent	409.35	79.14	5.17	<0.001	1.38
RT neutral	383.37	72.37	5.30	<0.001	1.39
RT incongruent	477.99	93.44	5.12	<0.001	1.37

*Age effect on the three conditions of Stroop task. RT, reaction time.*

**TABLE 6:** Relation b_n_ on mediation analysis.

	Coeff	Se	*t*	*p*	Std
**RT congruent**					
TST IP to AP	0.37	0.83	0.44	0.66	0.24
TST AP to IP	0.29	0.73	0.40	0.69	0.18
RT IP to AP	−0.18	0.25	−0.75	0.46	−0.36
RT AP to IP	0.14	0.15	0.95	0.35	0.46
RevT IP to AP	−0.23	0.25	−0.90	0.38	−0.44
RevT AP to IP	0.06	0.17	0.39	0.70	0.19
**RT neutral**					
TST IP to AP	0.10	0.81	0.12	0.91	0.06
TST AP to IP	0.70	0.71	0.97	0.34	0.39
RT IP to AP	−0.27	0.24	−1.13	0.27	−0.49
RT AP to IP	−0.30	0.15	−2.03	0.05	−0.88
RevT IP to AP	−0.19	0.25	−0.78	0.44	−0.34
RevT AP to IP	−0.15	0.16	−0.95	0.35	−0.43
**RT incongruent**					
TST IP to AP	−0.30	0.50	−0.61	0.55	−0.22
TST AP to IP	−0.82	0.44	−1.87	0.07	−0.59
RT IP to AP	*0.34*	*0.15*	*2.33*	*0.03*	*0.79*
RT AP to IP	*0.21*	*0.09*	*2.32*	*0.03*	*0.78*
RevT IP to AP	*0.33*	*0.15*	*2.16*	*0.04*	*0.74*
RevT AP to IP	0.16	0.10	1.67	0.11	0.58

*Predictions of the switching scores from the three Stroop task conditions. Italic: signitificant results. TST, total switching time; RT, reaction time; RevT, reversal time; IP, in-phase; AP, anti-phase.*

**TABLE 7:** Bootstrap analyses of the indirect “ab_n_” paths.

	Effect	BootSE	BootLLCI	BootULCI	Standardized
RT IP to AP	163.53	97.90	–65.66	308.84	1.08
RT AP to IP	99.34	42.25	31.25	200.40	1.07
RevT IP to AP	156.31	103.13	–83.79	307.15	1.01

*RT, reaction time; RevT, reversal time; IP, in-phase; AP, anti-phase.*

## Discussion

The present study aimed to determine whether inhibition processes are involved in age-related changes in intentional switching between preferred coordination patterns. To achieve this objective, in addition to the bimanual switching tasks, participants performed Stroop tasks. Response times measured in the Stroop tasks and switching times measured in the bimanual coordination task were analyzed through correlation and mediation analyses to assess the involvement of inhibition processes in bimanual switching. In the bimanual coordination tasks, RTs were hypothesized to be related to dismantling ongoing pattern and related to triggering the transition, while RevT was related to the onset of the effective transition. Our main hypotheses were that: (i) RT and RevT would be more directly sensitive to age-related alteration of inhibition processes; (ii) TST would be prominently related to intrinsic dynamics that is, to the overall stability of the intrinsic coordination patterns.

### Effects of Age, Frequency, and Coordination Mode on Pattern Stability

First of all, we analyzed the effects of the different dependent variables on pattern stability. Results showed that pattern stability was sensitive to age, oscillation frequency, and coordination modes. Specifically, on the one hand, the IP coordination pattern was more stable than the AP one in both young and older adults. On the other hand, older adults were less stable than young participants, whatever the coordination mode, excepted at the highest frequency. Thus, the effect of frequency on pattern stability was not systematic in older adults. This result can be explained by the small difference observed between the chosen lower frequency and the transition frequency in older adults (mean difference ±0.25 Hz in older and ±0.75 Hz in young participants, respectively). Accordingly, we averaged the two frequencies in the subsequent analyses. Then, as currently observed in previous studies, the AP pattern was less stable than the IP one in both young (Kelso, 1984; Temprado et al., 2010) and older adults (Greene and Williams, 1996; Temprado et al., 2010). This observation was a prerequisite to assess how pattern stability affected switching times. In addition, the SD of the RP was larger in older adults than in young adults, for both the IP and the AP pattern. Age-related increase in behavioral variability is consistent with current observations made in numerous cognitive and motor tasks. It is currently attributed to an age-related increase in neural noise (e.g., Li et al., 2001; Hultsch and MacDonald, 2004). However, in bimanual coordination tasks, the consequences of increased neural noise on behavioral variability might be magnified by an age-related decrease in bimanual coupling strength (see Haken et al., 1985 for a theoretical account and Temprado et al., 2010 for consistent results). In brain activation literature, Globe et al. find out that compared to young adults, older adults showed increased activity during the AP as compared to the IP coordination mode in several brain areas typically ascribed to more cognitive aspects of performance. For instance, the greater bilateral activations found in inferior frontal gyri, for older adults subjects during bimanual coordination might also reflect a cognition-based difference between young and old subjects.

### Effects of Age, Frequency, and Coordination Mode on Switching Response Times

We analyzed the effects of the different dependent variables on TST. Results showed that switching time was slower in older than in young adults. This finding is consistent with those observed in previous studies (Greene and Williams, 1996; Coxon et al., 2010). However, this effect was modulated by oscillation frequency and coordination patterns. In young adults, TST was sensitive to the direction of switching at the highest frequency. Specifically, it was shorter for AP to IP than for IP to AP. Thus, the results observed in young adults were roughly consistent with those reported in previous studies (Carson et al., 1996; De Luca et al., 2010). They confirmed that, at least at the highest frequency, switching time was related to intrinsic dynamics that is, to the stability of the initial pattern: the less stable the pattern (i.e., AP), the shorter the switching time (AP to IP direction). More surprising is the lack of effect of coordination mode and frequency on TST in older adults. This result is, however, consistent with those observed for pattern stability in the present study (i.e., no effect of frequency on pattern stability). It might be attributed to the small difference between the low and high frequencies used in older adults. This result led us to average the two frequencies in the subsequent analyses of switching response times.

In these subsequent analyses, results showed that TST was sensitive to age. The influence of intrinsic dynamics on TST was also observed as indicated by the nearly significant effect (i.e., *p* = 0.07) of the direction of switching: switching from AP to IP tended to be faster than switching from IP to AP. These results are consistent with those observed in previous studies (Greene and Williams, 1996; Coxon et al., 2010; De Luca et al., 2010). Conversely, RT and RevT were only affected by age. These results suggest that the first part of the switching process was prominently affected by age-related changes in cognitive processes, and few if any by pattern stability, while the second part (i.e., stabilization on the targeted pattern) was more specifically related to intrinsic dynamics. Correlation and mediation analyses confirmed this hypothesis.

### Effects of Age in Stroop Task

The results observed in the Stroop task showed that responses times were slower in older adults than in young adults, whatever the condition (i.e., congruent, neutral, and incongruent). Moreover, response times observed in the incongruent condition were longer than those observed in neutral and congruent conditions. These results are consistent with those observed in previous studies, which suggested that age-related increase in response times reflects decreased selective attention and response inhibition processes (e.g., Milham et al., 2002; Davidson et al., 2003; Troyer and Murphy, 2007).

### Mediation of Inhibition Processes in the Effects of Age and Coordination Modes on Stability and Switching Response Times

Correlation analyses showed that the stability of IP and AP patterns was differently correlated with response times in the Stroop task.

Specifically, while both IP and AP patterns correlated with processing speed, reflected by the congruent and neutral Stroop scores, inhibition seemed to only be involved in the AP pattern, because the incongruent Stroop response time only correlated with AP. These results suggest that in addition to requiring more attention (e.g., Temprado et al., 1999; Monno et al., 2000), maintaining the less stable pattern involves more strongly inhibition processes than maintaining the more stable, IP pattern. Conversely, the stability of the IP pattern seems to be more related to the general efficiency of information processing in the neuro-behavioral system than on inhibition processes. To our knowledge, it is the first time that experimental evidence is given in support of the strong involvement of inhibition processes in performing the AP pattern. This hypothesis is consistent with the HKB model of coordination dynamics (Haken et al., 1985), which assumes that the IP pattern is a spontaneous pattern that involves few cognitive/attentional processes, while the AP pattern requires additional cognitive involvement to preclude destabilization and to avoid spontaneous transition to the IP pattern (e.g., Temprado et al., 1999; Monno et al., 2000).

Correlation analyses also showed relations between switching times and response times observed in the Stroop task. First of all, results showed that, for both IP to AP and AP to IP directions, RT and RevT were both positively correlated with response times observed in the incongruent condition. This result suggests that the early stage of switching (which requires a dismantling of the ongoing pattern) is related to inhibition processes for both directions of switching.

It is noticeable that different results were observed for the correlation between TST and response times in Stroop tasks. Indeed, for the IP to AP direction, TST was not correlated with responses times in Stroop task, while for the AP to IP direction, it was correlated with congruent and neutral conditions. This result suggests that, at least in the AP to IP direction, the entire process of switching is related to global information processing efficiency of the neuro-behavioral system.

Mediation analyses give credence to these hypotheses. Indeed, “path a” of the mediation analysis (from age to response times in Stroop task) showed a large size effect in the three Stroop conditions (congruent, neutral, and incongruent). “Path b” of the mediation analysis (from Stroop task to switching response times) showed a significant relation between response times in the incongruent condition and RT in both directions (IP to AP and AP to IP). In addition, a significant relation was also found between response time in the incongruent condition and RevT in the IP to AP direction. The analysis of indirect mediation effect of the relationship between age and RT in the incongruent condition showed that the effect of age on the RT of AP to IP direction was mediated by performance on Stroop incongruent condition, with wide effect size (>1). In addition, 92.5% of the effect of age on the RT of AP to IP transition disappeared when mediators were taken into account. This result is consistent with those observed for the stability of coordination patterns (i.e., the correlation between Stroop task and SD of the RP of the AP pattern).

Thus, in summary, the results of the present study suggest that bimanual switching is associated with inhibition processes, at least in the AP to IP direction. However, the mediation of the cognitive processes involved in the incongruent condition of the Stroop task seems to be more specific to the AP to the IP direction of switching. Accordingly, it can be concluded that switching from AP to IP strongly involved inhibition processes. This hypothesis is consistent with the results observed by Heuninckx et al. (2005), who showed that in complex coordination tasks, the older adults exhibited additional activation in anterior rostral cingulate and dorsolateral prefrontal cortex, which is known to be involved in suppression of preponent response tendencies and inhibitory cognitive control.

However, the present results may seem, at first sight, counterintuitive. Indeed, it has been shown in previous studies that (i) switching time is related to the stability of the ongoing pattern (e.g., Scholz and Kelso, 1990) and (ii) during the transition, the involvement of BG is lower for AP to IP than for IP to AP (De Luca et al., 2010). On the basis of these results, Kelso (2012) formalized that inhibition processes are less involved once the transition started when switching from the less stable to the more stable pattern (i.e., AP to IP). A different picture is, however, observed for RT, which corresponds to dismantling the ongoing pattern to trigger the switch. Indeed, according to our results, it seems more costly to get out the AP attractor to switch to the IP pattern than vice versa since more attention/inhibition processes are involved in maintaining the AP pattern before the transition.

Another important result is that the entire process of switching seems to be rather related to information processing efficiency, at least for the AP to IP direction. Overall, both correlation and mediation analyses confirm the highly executive aspect of the bimanual coordination switching task, at least to trigger the early phase of switching (i.e., to get out the attractor and to dismantling the ongoing pattern).

These results can be related to those reported in the brain imaging literature. For instance, in a study on intentional switching in young adults, De Luca et al. (2010) observed that BG played a critical role in both the selection and maintenance of intentional behavior. Presumably, BG enacted a broad inhibition of all patterns coupled with focused disinhibition of the desired one. As a consequence, transient disruption of major cortical BG targets [i.e., supplementary motor area (SMA) and premotor cortex (PMC)] induced spontaneous switching from less to more stable movement patterns, but not the reverse. Accordingly, one interpretation holds that thanks to the mediation of BG and its connected cortical circuitry, longer switching time is required to destabilize the more stable pattern and simultaneously stabilize the less stable one. In other words, as predicted by the theory of coordination dynamics, intention exerted through BG circuits both acts upon and is constrained by the intrinsic dynamics of coordination patterns (e.g., Kelso, 2012). In support of this interpretation, Coxon et al. (2010) proposed that longer switching time observed in older adults reflects the fact that older adults are less successful than young ones in recruiting an inhibitory BG circuit needed to temporarily decouple hands and to promote an alternative coordination mode. However, Coxon et al. (2010) also observed that the elderly used an alternative strategy to select the required movement pattern, as indicated by increased activation of the prefrontal cortex. These findings might reflect the additional cognitive effort required to switch to the less stable and more attention-demanding AP coordination pattern (Temprado et al., 1999).

Additionally, when inhibitory processes are studied in a stricter sense by means of inhibition tasks that require overcoming or stopping a planned go-response, it appears that a set of particular brain areas is prominently involved [right inferior frontal cortex, (pre)SMA and BG (including striatum but also STN)]. If one considers work on switching behavior during bimanual coordination and stopping behavior (Coxon et al., 2010), it can be witnessed that the brain regions involved in these two types of behaviors are partly shared in young and older adults. In other words, the fact that brain regions involved in outright inhibition of action and switching are partly shared suggests that part of switching does require recruitment of inhibitory processes as mediated by the aforementioned brain regions. On top of that, older adults will additionally recruit prefrontal brain regions to support complex behaviors such as switching^1^.

## Conclusion

The results of the present study are consistent with previous observations made in bimanual coordination tasks, confirming that cognition both acts upon the intrinsic coordination dynamics and is constrained by it (Greene and Williams, 1996; Temprado et al., 1999; Coxon et al., 2010; De Luca et al., 2010). Accordingly, when considered within groups, switching from the less stable to the most stable pattern (i.e., AP to IP) incurs a higher cognitive load than switching in the opposite direction. This is consistent with the predictions of the HKB model of coordination dynamics (Scholz and Kelso, 1990; Kelso, 2012). On the other hand, when considered between groups, switching is more costly for both directions. In particular, contrary to the predictions of the theory of coordination dynamics, switching time from AP to IP was longer in older than in young participants.

Our results also show that age-related alterations of bimanual switching are mediated by inhibition processes. Specifically, the mediation through inhibition processes was stronger when participants were required to switch from AP to IP than in the opposite direction. Further investigation should be done to explore how training of EFs (e.g., selective attention and inhibition processes) might improve performance in bimanual switching tasks in older adults.

## Data Availability Statement

The datasets generated for this study are available on request to the corresponding author.

## Ethics Statement

The studies involving human participants were reviewed and approved by the Local Ethics Committee of Aix-Marseille University. The patients/participants provided their written informed consent to participate in this study.

## Author Contributions

J-JT, MT, and AL wrote the first draft of the manuscript. J-JT, MT, MJ-V, LD-R, and RS-M contributed to data recording and data analysis. All the authors contributed to critically revising the manuscript. They gave final approval of the version to be published and agreed to be accountable for all aspects of the work.

## Conflict of Interest

The authors declare that the research was conducted in the absence of any commercial or financial relationships that could be construed as a potential conflict of interest.

## Footnotes

1 We thank the reviewer for his suggestion.
